# “Good and bad investments” in public health stocks amid the COVID-19 shock: evidence from a transformer-based model

**DOI:** 10.3389/fpubh.2025.1644055

**Published:** 2025-10-28

**Authors:** Dezhi Zhao, Yanguo Li, Ruitao Gu

**Affiliations:** ^1^School of Economics, Yunnan University of Finance and Economics, Kunming, China; ^2^School of Finance, Yunnan University of Finance and Economics, Kunming, China

**Keywords:** public health crisis, public health stocks, transformer model, return prediction, investments

## Abstract

**Introduction:**

Major public health emergencies have profoundly reshaped the risk structure and resource allocation logic of capital markets. The market performance of public health-related enterprises has exhibited substantial heterogeneity across different stages of the pandemic, characterized by both considerable risks and emerging opportunities. Understanding this dynamic process is essential for maintaining financial stability and promoting rational investment behavior.

**Methods:**

Using the COVID-19 pandemic as the research background, this study selects 55 constituent stocks from the China Securities Index (CSI) Public Health Index as the research sample. A deep learning model based on the Transformer architecture is employed to forecast stock returns and construct long-short investment portfolios. By conducting stage-wise comparisons spanning the pre-pandemic period, the initial outbreak, the normalization phase, and the post-pandemic era, the study reveals the profound temporal evolution and dynamic impacts of public health crises on market investment behavior.

**Results:**

The empirical results reveal that the capital market underwent substantial structural reshaping during the initial phase of the pandemic. The Transformer model effectively identified excess return signals from healthcare and epidemic-prevention enterprises, thereby achieving outstanding investment performance. In the mid-pandemic stage, increased market volatility and policy uncertainty weakened the model's stability. As the market transitioned into the post-pandemic period, rationality gradually returned. Similar to the pre-pandemic stage, firms' performance became increasingly driven by fundamentals rather than policy influences, leading to a marked improvement in the model's predictive accuracy and screening capability.

**Conclusion:**

This study systematically reveals the structural differentiation and dynamic evolution of public health-related enterprises during the pandemic, thereby extending the research frontier at the intersection of public health emergency response, financial risk, and investment portfolio construction. By bridging these domains, it provides both theoretical foundations and empirical evidence to guide investment strategies and policy formulation for industries closely associated with public health, contributing to more resilient and informed financial decision-making amid future crises.

## 1 Introduction

Since its outbreak at the end of 2019, the COVID-19 pandemic has rapidly escalated into a global public health crisis, exerting unprecedented pressure on healthcare systems, economic structures, and capital markets worldwide. The pandemic has profoundly affected all aspects of society, prompting governments to implement emergency measures such as lockdowns and quarantines. These interventions severely disrupted global supply chains and impacted nearly every industry sector (1, 2). However, the degree of resilience across industries during the pandemic has varied significantly (3). For example, during the crash of the U.S. stock market, sectors such as natural gas, healthcare, and software generated substantial positive returns, whereas sectors like oil, real estate, and entertainment experienced sharp declines in valuation (4).

Beyond its profound economic repercussions, the COVID-19 pandemic has reshaped social structures and market mechanisms, fundamentally altering daily life, investor behavior, and market expectations. As sectors closely tied to pandemic prevention and control, public health-related industries have not only endured severe disruptions but also presented significant market opportunities, emerging as a focal point in capital markets. On one hand, governments around the world have increased support for these industries, aiming to enhance structural resilience and improve preparedness for future public health emergencies. On the other hand, fluctuations in industry stock prices not only reflect changes in firms' fundamentals but also influence investors' asset allocation decisions and a country's broader macroeconomic conditions. Therefore, accurate forecasting of stock price trends is essential—not only for enabling investors to make informed investment decisions, but also for supporting governments in conducting macroeconomic oversight based on sectoral stock price dynamics (5).

The price dynamics of public health-related stocks reflect not only corporate performance and investor risk preferences but also signal macroeconomic policy orientations and industry development expectations. While sub-sectors such as medical services, vaccine development, biomedical testing, pharmaceutical manufacturing, and digital health have drawn considerable market attention due to their close ties to pandemic prevention and control, it is important to recognize that despite the sector's overall stage-specific investment potential, firms within it exhibit substantial heterogeneity in both performance dynamics and structural characteristics. On one hand, companies whose products directly support epidemic prevention efforts—such as vaccines, diagnostic testing, and protective equipment—have achieved notable revenue growth and market capitalization expansion, emerging as high-quality targets for investors during the pandemic. Liu et al. (6) found that in the U.S. market, biotechnology and healthcare stock indices were positively correlated with the severity of the pandemic, suggesting that these assets could effectively supplement the burden on the healthcare system. On the other hand, some firms have faced operational challenges due to sudden shifts in demand, labor shortages, and supply chain disruptions (7), resulting in declining profitability and downward pressure on stock prices. The pandemic has not only exposed systemic risks but has also catalyzed structural adjustments and technological innovation within the industry, rendering the public health sector increasingly complex and dynamically evolving. The heterogeneity of public health-related stocks presents investors with heightened uncertainty and risk management challenges, while also requiring policymakers to monitor market signals as indicators of industry operations and policy effectiveness. Sun et al. (8) specifically analyzed medical sector portfolios during the pandemic, and Esparcia and López (9) investigated the pharmaceutical industry in conjunction with the healthcare sector from a portfolio investment perspective.

Moreover, across the pre-pandemic, pandemic, and post-pandemic stages, public health-related enterprises have exhibited markedly different impact trajectories, recovery dynamics, and market feedback mechanisms. This temporal heterogeneity offers an ideal quasi-natural experimental setting for the academic exploration of investment strategies, corporate behavior, capital market responses, and the evolution of policy interventions. It encompasses dynamic variations across different phases of the pandemic, including virus transmission, policy implementation, and sectoral adjustment. Particularly in financial markets, stock prices—serving as composite indicators of corporate fundamentals, industry outlooks, and investor sentiment—render the public health sector a critical lens through which the economic transmission mechanisms of the pandemic can be observed. Accordingly, from the perspectives of both investors and policymakers, systematically identifying the market performance patterns of public health enterprises throughout the progression of the pandemic and uncovering their underlying drivers is of significant importance. Such insights contribute not only to a deeper understanding of the sector but also to the optimization of investment strategies and the refinement of policy instruments.

However, traditional econometric models are often built on linear assumptions, making them inadequate for capturing the nonlinear and complex dynamic patterns inherent in firm performance—particularly when dealing with high-frequency, non-stationary financial data. In recent years, with the advancement of artificial intelligence technologies, machine learning and deep learning algorithms have been increasingly applied to interdisciplinary research spanning public health, finance, and economics. Some studies by Gao et al. (10), Vaughan et al. (11), Bassiouni et al. (12), Alzaman (13), and Abedin et al. (14) have successfully employed deep learning methods in these domains. Moreover, numerous studies have demonstrated that machine learning methods exhibit significantly superior predictive capabilities compared to traditional econometric models (15). Among these, the Transformer model—characterized by its attention mechanism—has garnered significant attention in financial forecasting and economic analysis due to its exceptional sequence modeling capabilities and powerful multidimensional feature extraction. Compared to traditional machine learning approaches, the Transformer exhibits marked advantages in handling high-dimensional, dynamic, and complex time series data, making it particularly suitable for modeling high-frequency and strongly non-stationary financial series. Wang et al. (16) highlight that the Transformer's deep learning architecture overcomes limitations of conventional models such as CNN and RNN, leading to its widespread adoption across various disciplines. Compared with the sequential nature of RNN and LSTM, the self-attention mechanism of Transformers enables parallel training and facilitates the capture of global contextual information more effectively.

Against this backdrop, this study adopts an investment analysis perspective and develops a deep learning forecasting framework based on the Transformer model to model the stock price sequences of public health-related listed companies in China under the impact of the COVID-19 pandemic. The framework systematically identifies firms' dynamic market performance and explores its intrinsic links with industry structure, policy factors, and market feedback mechanisms. By segmenting the pandemic timeline into pre-pandemic, individual pandemic years, and post-pandemic phases, the study constructs investment portfolios and conducts performance evaluations across different periods. In doing so, it seeks to uncover the evolutionary paths of sub-sector assets within the public health industry during the pandemic and provide practical strategic guidance for investors, while offering empirical support for policymakers in industry monitoring and intervention.

This paper makes three main contributions. First, from the perspective of investment heterogeneity, it systematically reveals the structural divergence and dynamic changes among public health-related firms during the pandemic. Second, it introduces the Transformer model into the investment portfolio construction process in a novel way, enhancing the forecasting accuracy for complex, nonlinear financial time series and providing a new approach to financial investment modeling. Third, by conducting empirical analyses across multiple disaggregated time intervals, the study extends the research frontier at the intersection of public health and finance, and offers both theoretical and empirical foundations for post-pandemic investment strategies and policy formulation in public health-related industries.

The structure of this paper is organized as follows: Section 2 presents a literature review, summarizing existing research on public health events, financial markets, sectoral heterogeneity, and deep learning models. Section 3 outlines the research methodology, including the architecture of the Transformer model, portfolio construction methods, and evaluation metrics. Section 4 details the empirical analysis and results. Section 5 concludes the study.

## 2 Literature review

The COVID-19 pandemic, as a global public health crisis, has profoundly transformed the functioning of the world economy and the structure of financial markets. Existing research has extensively examined the transmission channels and mechanisms through which the pandemic has impacted the global economy, highlighting that such sudden public health events are typically accompanied by a surge in information uncertainty (17), the spread of market panic (18), and heightened stock market volatility (19). A key characteristic of capital market responses during this period has been the pronounced performance divergence across industry sectors. For instance, sectors such as crude oil, entertainment, and hospitality suffered severe setbacks, whereas industries including medical equipment, software, and food experienced counter-cyclical growth (4). At the same time, travel restrictions and various epidemic control measures significantly deteriorated the stock market performance of tourism-related companies globally (20). The panic-driven consumption behavior induced by the pandemic also affected multiple sectors, prompting firms to adjust production and operations to cope with supply–demand imbalances in their product lines (21). Moreover, investor behavior exhibited heterogeneous patterns of risk contagion across regions, market conditions, and levels of development (22). Chu et al. (23) constructed a “pandemic transmission network” and quantified its risk scores, effectively predicting financial market interconnectedness and potential contagion. Franzolini et al. (24), using a dynamic Gaussian graphical model, captured structural shifts in the U.S. stock market and identified sharp changes in inter-sector linkages during the COVID-19 period.

Focusing specifically on public health-related enterprises, existing literature highlights significant structural heterogeneity in their responses to the pandemic. On one hand, companies directly involved in epidemic prevention—such as vaccine manufacturers, biomedical testing firms, and suppliers of protective equipment—gained substantial favor from capital markets due to their alignment with urgent healthcare demands. On the other hand, traditional healthcare service providers faced considerable challenges stemming from a decline in non-emergency visits, rising operational costs, and restrictions on personnel mobility. For example, Perroni et al. (25), in their study of Brazil's healthcare sector during the COVID-19 pandemic, noted considerable variation in stock performance across sub-industries, with pharmaceutical firms demonstrating greater resilience compared to drug distributors and medical service providers. Maleki and Ghahari (26) focused specifically on pharmaceutical stocks and found that their price fluctuations were influenced by multiple factors and evolved dynamically across different phases of the crisis. Wang et al. (5), in their analysis of Chinese pharmaceutical equities, identified significant interactions between investor sentiment, the severity of the pandemic, and stock price trends in the pharmaceutical sector. The survey conducted by Felt-Lisk et al. (27) further reveals the changes that occurred in healthcare services before and after the COVID-19 pandemic.

Meanwhile, corporate social responsibility (CSR) also emerged as a critical determinant of firm performance during the pandemic. Li et al. (28) found that variation in CSR practices influenced stock returns during the crisis period. From an ESG (Environmental, Social, and Governance) perspective, Zhou and Zhou (29) observed that firms with stronger ESG performance experienced significantly lower stock price volatility, suggesting that sound ESG practices enhance price stability. Krammer (30) argues that innovative firms tend to be more attuned to changes in the external environment and are capable of reallocating assets and resources accordingly. Moreover, they typically exhibit a high level of managerial diligence, which proves particularly valuable in responding to global crises such as COVID-19. Liu et al. (31) employed machine learning methods to quantify the impact of public health emergencies on small and medium-sized enterprises (SMEs), finding that the adverse effects varied significantly across industries. Within the healthcare sector, for example, firms overly dependent on innovation, advertising, or environmental sustainability experienced lower profitability during the crisis, whereas those emphasizing personal sales and social and governance sustainability demonstrated greater resilience and financial performance (32).

This heterogeneity is not static but evolves dynamically with different stages of the pandemic—such as outbreak, spread, mitigation, and recurrence—as well as adjustments in policy responses including lockdowns, stimulus measures, and the resumption of economic activities. Understanding the dynamic behavior and valuation shifts of public health enterprises under pandemic shocks from an investment perspective has thus emerged as a new research frontier in recent years. In parallel, investor behavior during the pandemic has also displayed clear phase-specific characteristics. Prior studies have shown that asset pricing in capital markets during the pandemic has been influenced not only by macroeconomic fundamentals but also by investor attention (33), fear indices (34), and information asymmetry (35). These factors are particularly pronounced in the case of public health-related stocks. Many investors pursued short-term speculative opportunities in concept stocks linked to epidemic themes, resulting in a coexistence of speculative assets and firms with long-term intrinsic value. As a consequence, traditional financial indicators or static market capitalization rankings may fail to effectively identify high-quality investment targets. This underscores the urgent need for a dynamic identification mechanism capable of capturing time-series characteristics and nonlinear market relationships, thereby enabling investors to achieve more effective asset allocation under conditions of heightened uncertainty.

In terms of research methodology, although traditional approaches such as event studies, regression analysis, and panel data models were widely applied during the early stages of the pandemic, they exhibit notable limitations when dealing with high-frequency nonlinear data, non-stationary processes, and heterogeneous market structures. Ronaghi et al. (36) focused on the sudden and adverse effects of the COVID-19 outbreak, emphasizing that the pandemic has had a detrimental impact on econometrics and stock markets. They further argued that AI- and machine learning-based forecasting models, particularly those employing deep neural network architectures, may serve as critical tools in mitigating these negative effects. In recent years, the application of artificial intelligence—especially deep learning—has expanded rapidly, with deep learning methods becoming cutting-edge techniques for spatiotemporal prediction across various domains, including public health (37).

Numerous researchers have experimented with different deep learning architectures to model stock prices, volatility, sentiment indicators, and other financial variables, achieving promising results. For instance, Omar et al. (38) applied both random forest and deep neural network (DNN) models to forecast the KSE-100 index, finding that the DNN model performed well across the full time span and in the pre-pandemic period, while the RF model yielded better results during the pandemic. Sharaf et al. (39) proposed a stacked LSTM model integrated with news sentiment analysis, demonstrating that their hybrid system significantly enhanced stock prediction accuracy. Yang et al. (40) introduced a hybrid MEEMD-LSTM-MLP framework to analyze stock index forecasting during the pandemic, showing that the model outperformed benchmarks in both emerging and developed markets, and delivered strong predictive performance amid extreme market volatility triggered by COVID-19. Ray et al. (41) employed an innovative hybrid deep learning algorithm to study sectoral indices of Indian stocks, focusing particularly on pharmaceutical firms involved in vaccine development. Their model outperformed several baselines, especially during pandemic-induced market trends. Li et al. (42) further explored the integration of deep learning and reinforcement learning to develop highly adaptive trading strategies. More advanced models, such as Deep RankNet combined with genetic algorithm-based hyperparameter optimization, have also been proposed for asset selection and portfolio optimization, demonstrating superior performance over conventional approaches (13).

In recent years, Transformer models based on the attention mechanism have garnered increasing interest from researchers across diverse disciplines, owing to their breakthrough performance in natural language processing and time series forecasting. Transformer-based models have already been applied in COVID-19-related CT image analysis studies (43, 44). Wang et al. (16) demonstrated that the Transformer, through its encoder–decoder architecture and multi-head attention mechanism, outperforms conventional deep learning models in terms of predictive accuracy and net value analysis, and further emphasized that financial time series forecasting represents a particularly promising application domain for the Transformer architecture. Zhang et al. (45) implemented a transformer-based deep learning framework incorporating various attention mechanisms and integrated both textual and stock price data, validating the TEANet framework's effectiveness in predicting stock fluctuations. Kim et al. (46) enhanced the prediction accuracy of the S&P 500 index by combining FinBERT with LSTM. Gao et al. (47) utilized a Transformer-based architecture to extract investor-related features and employed a Gray Wolf Optimizer-enhanced SVR model to conduct quantitative investment research. Mishra et al. (48) developed a multi-transformer neural network model to forecast market volatility, highlighting the superior performance of hybrid neural networks. They found that models incorporating Transformer structures consistently outperformed standalone models—even under unpredictable conditions such as the COVID-19 pandemic.

In summary, Transformer remains a relatively novel deep learning architecture, with its applications only emerging in recent years. The use of Transformer models to identify industry dynamics and inform investment decisions under pandemic conditions remains an emerging research area—particularly when applied to public health-related enterprises, which lie at the core of pandemic response. Existing studies have largely focused on macro-level or sector-level analyses, with limited research dedicated to forecasting the performance of public health enterprises amid the dynamic progression of the pandemic or constructing corresponding investment portfolios. Therefore, constructing a deep learning forecasting framework centered on the Transformer architecture—combined with firm-level stock time series data—offers both theoretical innovation and practical relevance in tracing the structural evolution of public health enterprises under pandemic shocks from an investment perspective. The outbreak of the pandemic has underscored the limitations of traditional static investment strategies and linear modeling approaches in coping with high uncertainty and nonlinear disturbances. Deep learning models, particularly Transformer-based frameworks, demonstrate substantial practical value in forecasting public health stock performance and designing crisis-responsive investment strategies. From both policy and investment standpoints, such models enable the precise identification of “good” and “bad” investments, thereby facilitating more efficient capital allocation, enhancing the resilience of healthcare systems, and promoting stability in financial markets.

## 3 Methodology

### 3.1 Transformer model

To enhance the modeling capacity for financial time series forecasting, this study adopts the Transformer model—a deep learning architecture based on the attention mechanism (49). Originally developed for natural language processing tasks, the Transformer has gained widespread adoption in recent years across various domains, including financial market prediction and economic behavior modeling, due to its superior ability to capture long-range dependencies, parallel computation efficiency, and scalability (50). Unlike traditional methods based on recurrent neural networks (RNNs) or convolutional neural networks (CNNs), the Transformer relies entirely on self-attention mechanisms for feature extraction. This design enables it to capture global dependencies across any positions within the input sequence, thereby improving predictive accuracy while reducing training costs.

The overall structure of the Transformer model adopts a typical encoder—decoder architecture, consisting of multiple stacked layers of encoders and decoders. The encoder is responsible for processing the input sequence and extracting key features, while the decoder dynamically references the encoder's output during the generation process to complete the prediction task. In the original Transformer framework, the core component is the scaled dot-product attention mechanism (50). This mechanism computes similarity scores between the input query (Q), key (K), and value (V) vectors to derive attention weights, which quantify the relative importance of each position in the input sequence. These weights are then used to perform weighted aggregation of the input information. The computation is formally expressed as:


(1) $$\begin{array}{l} \text{Attention }\left(Q,K,V\right)=\text{softmax}\left(\frac{QK^{⊤}}{\sqrt{d_{k}}}\right)V \end{array}$$

In Equation 1, $Q\in {\mathbb{R}}^{n\times d_{k}}$ denotes the query matrix, $K\in {\mathbb{R}}^{n\times d_{k}}$ is the key matrix, and $V\in {\mathbb{R}}^{n\times d_{v}}$ represents the value matrix. The term *d*_*k*_ refers to the dimensionality of the key vectors and serves as a scaling factor to prevent gradient vanishing or explosion during training. A core advantage of the attention mechanism lies in its flexibility to model dependencies between any two positions in a sequence, particularly outperforming RNNs in capturing long-range interactions. To further enhance the model's ability to capture diverse feature representations, the Transformer introduces the concept of multi-head attention (49). This mechanism executes multiple self-attention operations in parallel, with each instance referred to as a “head.” Each head learns a distinct subspace representation of the input, allowing the model to extract different types of information simultaneously. The formulation of multi-head attention is as follows:


(2) $$\begin{array}{l} \text{MultiHead }\left(Q,K,V\right)=\text{Concat }\left({\text{head}}_{1},...,{\text{head}}_{h}\right)W^{O} \end{array}$$

(3) $$\begin{array}{l} \text{hea}{\text{d}}_{\text{i}}=Attention\;\left(QW_{i}^{Q},KW_{i}^{K},VW_{i}^{V}\right) \end{array}$$

In Equations 2, 3, $W_{i}^{Q}$,$W_{i}^{K}$,$W_{i}^{V}$ denote the learnable linear projection matrices for the *i*-th attention head, while *W*^*O*^ is the output projection matrix. The multi-head attention mechanism enables the model to attend to the structural properties of the sequence from multiple perspectives and dimensions, thereby enhancing its expressive capacity. Both the encoder and decoder of the Transformer consist of multiple stacked layers. Each layer contains two primary subcomponents: the multi-head self-attention mechanism and a position-wise feedforward neural network (FFN). Residual connections and layer normalization are applied between each sublayer to improve training stability and facilitate efficient gradient propagation. The position-wise feedforward network independently transforms each position in the sequence and is defined as follows:


(4) $$\begin{array}{l} \text{FFN}\left(\text{x}\right)=\max\left(0,xW_{1}+b_{1}\right)W_{2}+b_{2} \end{array}$$

In Equation 4, $W_{1}\in {\mathbb{R}}^{d_{model}\times d_{ff}}$ and $W_{2}\in {\mathbb{R}}^{d_{ff}\times d_{model}}$, where *d*_*model*_ denotes the model's embedding dimension and *d*_*ff*_ is the hidden dimensionality of the feedforward layer. As the Transformer architecture lacks an inherent mechanism for modeling sequential order, positional encoding is introduced to explicitly incorporate temporal information into the input representations. In this study, we adopt a fixed positional encoding approach, which is defined as follows:


(5) $$\begin{array}{l} \text{P}{\text{E}}_{\left(\text{pos},2\text{i}\right)}=\sin\left(\frac{pos}{10000^{2i/d_{\text{model}}}}\right) \end{array}$$

(6) $$\begin{array}{l} \text{P}{\text{E}}_{\left(\text{pos},2\text{i}+1\right)}=\cos\left(\frac{pos}{10000^{2i/d_{\text{model}}}}\right) \end{array}$$

In Equations 5, 6, *pos* denotes the position index within the sequence, and *i* represents the dimension of the positional encoding vector. By incorporating sine and cosine functions in the encoding scheme, the model gains the ability to learn positional information, thereby enabling temporal awareness and supporting generalization to sequences of varying lengths. This approach also facilitates the modeling of positional relationships during prediction. Compared to traditional sequential models, the Transformer exhibits several distinct advantages: (1) Highly parallelizable computation: By eliminating the need for serial dependency across time steps, the Transformer significantly improves training efficiency. (2) Enhanced long-range dependency modeling: the attention mechanism enables full connectivity across all positions in the input sequence, making it more effective at capturing long-term interactions. (3) Modular and extensible architecture: each sublayer is independently structured, allowing for flexible integration with other models or the stacking of customized modules. (4) Versatility across tasks: the Transformer is well-suited for a wide range of applications, including time series forecasting, asset price modeling, and textual data processing. In summary, this study adopts the Transformer model as the core architecture for return prediction, integrating daily trading data from public health-related enterprises to construct a deep learning forecasting system that adapts to the evolving dynamics of different pandemic stages. This approach aims to enhance the model's responsiveness under structural regime shifts and improve the effectiveness of investment signal identification.

### 3.2 Long-short portfolio construction

In the field of asset pricing and investment strategy research, long-short portfolio construction is commonly employed to systematically assess the explanatory power of predictive signals for future returns. This study adopts the long-short framework, as utilized by Leippold et al. (51) in their investigation of stock market dynamics. The core principle of this approach lies in constructing a strategy that simultaneously goes long on assets with high ranking values and short on those with low ranking values. This design enables isolation and quantification of specific factor risk premia and has been widely applied in both academic and practical investment contexts. The basic procedure of the long-short strategy involves ranking the sample assets based on a predictive indicator—such as momentum, valuation, market capitalization, or model-forecasted returns—and then dividing them into several quantile-based groups. A long portfolio is formed using the assets in the top quantile (with high indicator values), while a short portfolio is constructed from the bottom quantile (with low indicator values). The return of the strategy is calculated as the difference between the returns of the long and short portfolios. This method helps to neutralize the impact of market-wide systematic risk, thereby isolating the return contribution of the specific factor captured by the sorting variable.

At a given time point *t*, suppose the research sample includes *N* assets, each associated with a specific ranking indicator *S*_*i, t*_. The construction process of the long-short portfolio proceeds as follows: (1) Ranking stage: all sample assets are sorted in descending order according to their *S*_*i, t*_ values, yielding a ranked asset set {*i*_1_, *i*_2_, …, *i*_*N*_}. (2) Grouping: based on the ranking results, the assets are divided into several equally weighted or equally sized groups. Common grouping schemes include tertiles, quintiles, and deciles. In this study, we adopt the decile grouping method, whereby each group contains ~*N*/10 assets. (3) Construction of long and short portfolios: long portfolio: composed of the top 10% of assets with the highest indicator values, equally weighted. Short Portfolio: composed of the bottom 10% of assets with the lowest indicator values, also equally weighted. Long–Short Portfolio: a zero-cost strategy that takes a long position in the long portfolio and a short position in the short portfolio. The excess return of the strategy at time *t* + *1* is denoted as $R_{t+1}^{LS}$, calculated as follows:


(7) $$\begin{array}{l} R_{t+1}^{LS}=\frac{1}{n_{L}}\underset{i\in L}{\sum }R_{i,t+1}-\frac{1}{n_{S}}\underset{j\in S}{\sum }R_{j,t+1} \end{array}$$

In Equation 7, *L* and *S* represent the sets of assets in the long and short portfolios, respectively; *n*_*L*_ and *n*_*S*_ denote the number of assets in the long and short portfolios; *R*_*i, t* + 1_ and *R*_*j, t*+1_ refer to the returns of the *i*-th and *j*-th assets in period *t* + *1*, respectively. To capture the dynamic characteristics of portfolio performance across different periods, a rolling window approach can be employed. This involves updating the ranking indicators and reconstructing the portfolios at each time step, thereby generating a time series of long–short portfolio returns. Such a return series can then be used for subsequent return analysis, performance evaluation, and statistical significance testing.

Compared to purely long-only or short-only portfolios, the long—short strategy offers several key advantages: (1) Systematic risk hedging: by simultaneously incorporating both buy and sell positions, the strategy partially offsets the impact of overall market fluctuations. (2) Enhanced predictive power: by comparing assets in extreme quantiles, the approach amplifies the discriminatory power of the ranking indicator in forecasting future returns. (3) Broad applicability: the long—short framework is well-suited for testing various financial indicators—such as momentum, sentiment, valuation, and predicted returns—and is widely used in quantitative stock selection and strategy development. Accordingly, this study employs stock-level predicted returns generated by the Transformer model as the basis for ranking, and systematically constructs long—short investment portfolios. This allows for an evaluation of the model's practical utility and investment guidance value across different stages of the COVID-19 pandemic.

### 3.3 Evaluation metrics for return forecasting

To comprehensively assess the performance of the model in the task of financial time series prediction, this study adopts three commonly used error-based metrics as evaluation criteria: mean absolute error (MAE), mean squared error (MSE), and root mean squared error (RMSE) (52). These metrics capture the deviation between predicted and actual values from different perspectives and are widely recognized for their interpretability and general applicability. The Mean Absolute Error (MAE) measures the average magnitude of errors between predicted and true values, without considering their direction. It is formally defined as:


(8) $$\begin{array}{l} \text{MAE}=\frac{1}{n}\overset{n}{\underset{i=1}{\sum }}|{\hat{y}}_{i}-y_{i}| \end{array}$$

In Equation 8, ŷ_*i*_ represents the model's predicted value, *y*_*i*_ denotes the actual observed value, and *n* is the sample size. The MAE quantifies the average level of prediction error and shares the same unit as the original data, making it intuitively interpretable. Compared to squared-error-based metrics, MAE is less sensitive to outliers, making it particularly suitable for scenarios where volatility exists in the data but extreme values are not intended to dominate the error assessment. The mean squared error (MSE) measures the average of the squared differences between actual and predicted values. It is formally defined as:


(9) $$\begin{array}{l} \text{MSE}=\frac{1}{n}\overset{n}{\underset{i=1}{\sum }}{\left({\hat{y}}_{i}-y_{i}\right)}^{2} \end{array}$$

The MSE penalizes larger prediction errors more heavily, making it particularly valuable in scenarios where sensitivity to substantial deviations is important. However, its dimensionality is the square of the original unit, which limits its interpretability compared to MAE. The root mean squared error (RMSE) is defined as the square root of the MSE and is expressed mathematically as:


(10) $$\begin{array}{l} \text{RMSE}=\sqrt{\frac{1}{n}\overset{n}{\underset{i=1}{\sum }}{\left({\hat{y}}_{i}-y_{i}\right)}^{2}} \end{array}$$

RMSE retains the sensitivity of MSE to large prediction errors, while maintaining the same unit as the original data, thereby balancing interpretability and penalization. In model evaluation, RMSE is often used in conjunction with MAE for comparative analysis. A substantial discrepancy between the two may indicate the presence of significant outliers or extreme deviations in the prediction errors. As in Equations 9, 10, ŷ_*i*_ denotes the predicted value, *y*_*i*_ represents the actual observed value, and *n* is the total number of samples. In summary, this study employs a comprehensive evaluation framework using MAE, MSE, and RMSE to quantify prediction errors from multiple perspectives. This triad of metrics enhances the accuracy and robustness of model assessment, providing a more complete picture of the model's performance in financial forecasting tasks.

### 3.4 Portfolio performance evaluation metrics

To comprehensively evaluate the performance of investment portfolios across different market phases, this study introduces three annualized performance metrics from both return and risk-adjusted perspectives: annualized average return, Sharpe ratio, and Sortino ratio. These metrics not only capture the portfolio's long-term wealth accumulation potential but also reflect the structure of return volatility and downside risk. As such, they provide effective quantitative benchmarks for assessing the quality of investment strategies and are particularly valuable for evaluating robustness and risk control. The Annualized Return measures the compound growth of an investment over the entire holding period and serves as a fundamental and intuitive performance indicator. Given a portfolio's cumulative return *R*_*cum*_ over *T* trading periods (typically days), the annualized average return is defined as follows:


(11) $$\begin{array}{l} R_{annual}={\left(1+R_{cum}\right)}^{\frac{N}{T}}-1 \end{array}$$

In Equation 11, *N* represents the number of trading periods in a year (set to 252 in this study based on daily data), and *T* denotes the actual investment horizon. By standardizing the time dimension, this metric facilitates horizontal comparisons across strategies with different holding periods and reflects the long-term return potential of a portfolio under compounding conditions. The Sharpe Ratio evaluates the excess return earned per unit of total risk and is a classical measure for assessing the risk-adjusted performance of an investment portfolio. Its annualized form is defined as:


(12) $$\begin{array}{l} Sharpe\;Ratio=\frac{{\bar{R}}_{p}-R_{f}}{\sigma_{p}}\times \sqrt{N} \end{array}$$

In Equation 12, ${\bar{R}}_{p}$ denotes the average return of the portfolio, *R*_*f*_ represents the risk-free rate, and in this study, following the approach of Zheng-yang et al. (53), it is set to zero (*R*_*f*_ = 0). σ_*p*_ indicates the standard deviation of portfolio returns, and *N* is the annualization factor. A higher Sharpe ratio implies greater excess return per unit of risk, indicating better risk-adjusted performance. This metric is particularly suitable for evaluating strategies with relatively symmetric return distributions and controllable volatility. However, given that financial returns often exhibit skewness and fat tails, the Sharpe ratio's assumption of treating positive and negative volatility equally may underestimate actual risk exposure. Therefore, this study further introduces the Sortino ratio as a complementary risk-adjusted return metric. The Sortino ratio is an enhancement of the Sharpe ratio, accounting only for downside risk—i.e., deviations below the risk-free rate. It is defined as follows:


(13) $$\begin{array}{l} Sortino\;Ratio=\frac{{\bar{R}}_{p}-R_{f}}{\sigma_{d}}\times \sqrt{N} \end{array}$$

(14) $$\begin{array}{l} \sigma_{d}=\sqrt{\frac{1}{T}\overset{T}{\underset{t=1}{\sum }}\min{\left(R_{p,t}-R_{f},0\right)}^{2}} \end{array}$$

In Equations 13, 14, ${\bar{R}}_{p}$ denotes the average return of the investment portfolio, *R*_*f*_ is the risk-free rate, and *N* represents the annualization factor. The term σ_*d*_ refers to the downside deviation, which captures the standard deviation of returns falling below the risk-free rate by computing the squared deviations and averaging only over the negative return observations. This emphasizes the portfolio's ability to manage downside risk. A higher Sortino ratio indicates that the portfolio achieves greater excess return while being exposed to limited downside risk. The Sortino ratio is particularly sensitive to capital preservation and stable returns, making it especially appropriate for investors with low risk tolerance—such as those active in extreme market environments like public health crises. In summary, this study employs a three-dimensional performance evaluation framework—comprising annualized return, Sharpe ratio, and Sortino ratio—to assess the long–short portfolios from the perspectives of total return generation, risk-adjusted efficiency, and downside risk control. This framework facilitates robust comparisons across time periods and investment strategies, thereby enhancing the credibility of the empirical findings and their relevance for policy recommendations. It is especially well-suited for examining the risk–return reallocation patterns triggered by structural shifts in financial markets under the influence of public health emergencies.

## 4 Empirical analysis and results

### 4.1 Research data

This study investigates the capital market performance of public health-related enterprises from the perspective of shocks induced by sudden public health emergencies. Specifically, we select the constituent stocks of the China Securities Index (CSI) Public Health Index (Index Code: 931513) as the research sample. Abbreviated as “Public Health,” this index is composed of listed companies that are deeply engaged in public health-related sectors. It includes securities of firms involved in infectious disease prevention and control, medical equipment and protective consumables, healthcare information system development, and public health environmental governance. The index aims to reflect the overall market performance and development trends of listed companies focused on public health themes. In the area of pharmaceuticals and biotechnology related to infectious disease prevention and control, the index covers companies engaged in antibiotics, antiviral drugs, vaccines, diagnostic reagents, genetic engineering, and blood products. In the field of public health-related medical equipment and protective consumables, constituent companies include manufacturers of intensive care units (ICU) equipment, masks, medical gloves, and other relevant devices and consumables. For the development of healthcare information systems, the index incorporates firms involved in medical information systems and online diagnostic services. Meanwhile, in the field of public health environmental management, the index includes companies producing pollution detection equipment, medical waste treatment systems, and wastewater treatment technologies. The establishment of the CSI Public Health Index not only provides investors with a practical tool to monitor developments in the public health sector, but also offers policymakers valuable insights for strengthening public health infrastructure and optimizing resource allocation.

Considering data completeness and availability, this study selects daily data spanning from March 2, 2012, to March 7, 2025, to construct the analysis sample. After excluding firms with missing data, trading suspensions, or delistings that prevent inclusion in the study, a final sample of 55 publicly listed companies from the CSI Public Health Index—each closely related to the public health sector—was identified as the research target. For each stock, eight technical and trading features are used as input variables: opening price, closing price, highest price, lowest price, price change, trading volume, trading value, and turnover rate. The output variable is the daily return, which serves as the prediction target for the Transformer model. Descriptive statistics for the selected features are presented in Table 1.

**Table 1:** Descriptive statistics.

**Statistic**	**Open price**	**Close price**	**High price**	**Low price**	**Price change**	**Trading volume**	**Trading value**	**Turnover rate**	**Return**
Mean	2.1033E+01	2.1052E+01	2.1463E+01	2.0638E+01	1.1348E−02	1.4655E+07	2.4262E+08	1.7089E+00	3.1197E−04
Std. error	7.9333E−02	7.9418E−02	8.1015E−02	7.7734E−02	2.8834E−03	5.6528E+04	1.1650E+06	5.0382E−03	7.1158E−05
Median	1.4200E+01	1.4210E+01	1.4470E+01	1.3950E+01	0.0000E+00	7.9425E+06	1.0858E+08	1.0814E+00	0.0000E+00
Mode	7.0000E+00	7.0800E+00	7.1500E+00	7.2000E+00	0.0000E+00	2.6250E+06	1.3134E+07	4.5340E−01	0.0000E+00
Std. deviation	3.2443E+01	3.2478E+01	3.3131E+01	3.1789E+01	1.1792E+00	2.3117E+07	4.7643E+08	2.0603E+00	2.9100E−02
Variance	1.0525E+03	1.0548E+03	1.0976E+03	1.0105E+03	1.3904E+00	5.3439E+14	2.2698E+17	4.2450E+00	8.4678E−04
Kurtosis	1.0934E+02	1.0942E+02	1.0833E+02	1.1014E+02	2.1553E+02	1.3484E+02	9.8747E+01	3.4912E+01	3.7711E+01
Skewness	8.8612E+00	8.8611E+00	8.8190E+00	8.8907E+00	−2.0304E−01	7.6871E+00	7.6172E+00	4.5846E+00	−1.8368E+00
Range	6.8054E+02	6.7871E+02	6.8208E+02	6.6244E+02	9.3230E+01	9.8107E+08	1.5605E+10	3.9552E+01	9.0112E−01
Minimum	1.6700E+00	1.6800E+00	1.7000E+00	1.6600E+00	−5.0610E+01	3.6140E+03	1.5421E+05	3.3000E−03	−7.0000E−01
Maximum	6.8221E+02	6.8039E+02	6.8378E+02	6.6410E+02	4.2620E+01	9.8108E+08	1.5605E+10	3.9556E+01	2.0112E−01
Sum	3.5174E+06	3.5207E+06	3.5893E+06	3.4514E+06	1.8978E+03	2.4509E+12	4.0575E+13	2.8579E+05	5.2172E+01
Observations	1.6724E+05	1.6724E+05	1.6724E+05	1.6724E+05	1.6724E+05	1.6724E+05	1.6724E+05	1.6724E+05	1.6724E+05

The descriptive statistics in Table 1 reveal that price-related variables (opening, closing, high, and low prices) have an average value around 21, while the maximum exceeds 680 and the minimum is below two, indicating a highly stratified structure. Both skewness and kurtosis significantly deviate from normality, suggesting a widespread presence of extreme price observations in the sample. The mean of the price change is close to zero, with a standard deviation of 1.18; the maximum increase reaches 42.62, and the maximum decline is as large as 50.61, reflecting severe market adjustments during the pandemic shock. There is substantial disparity in trading volume and turnover amount, implying a high degree of heterogeneity in market activity across firms. The turnover rate exhibits right skewness, with notable occurrences of extreme high-frequency trading, indicating that market attention was concentrated on certain individual stocks. The mean return is 0.031%, with a standard deviation of 2.91%; skewness is −1.84, and kurtosis is 37.71, revealing fat tails and extreme negative return characteristics, which highlight systemic risk exposure during the public health crisis. Overall, public health-related firms in the sample period exhibit distinctive features of price dispersion, high volatility, and a high frequency of extreme events, indicating structural heterogeneity and market sensitivity under the impact of major health emergencies. These data characteristics provide a solid empirical foundation for subsequent model forecasting and investment strategy development.

Building on the above analysis, this study employs the Transformer model—known for its strong nonlinear modeling capabilities—to conduct return prediction. To improve both training efficiency and forecasting accuracy, the raw data must be properly preprocessed before being input into the Transformer model. Due to potentially large differences in the numerical scales of the original features, direct input into the model may lead to issues such as gradient instability, slow convergence during training, or even feature bias, thereby compromising model performance. To address this, the *Z*-score normalization method is adopted to transform the raw input features into a standardized format. This procedure aligns the data to a common scale and ensures consistency in numerical magnitude and distribution across features. The normalization is performed using the following formula:


(15) $$\begin{array}{l} x_{i}^{*}=\frac{x_{i}-\mu }{\sigma } \end{array}$$

In Equation 15, *x*_*i*_ represents the original feature value, μ is the sample mean of that feature, σ denotes the sample standard deviation, and $x_{i}^{*}$ is the standardized feature value. This normalization process helps to unify the numerical scale of input features, thereby improving the model's convergence efficiency and enhancing the transformer's stability and generalization ability when processing complex financial time series data.

### 4.2 Predictive performance across different time periods

To further examine the stage-specific impact of major public health events on the investment value and predictability of public health-related stocks, this study utilizes daily data from 55 constituent companies of the CSI Public Health Index over the full sample period from March 2, 2012, to March 7, 2025. The data are divided into five structurally significant sub-periods, each corresponding to a distinct market environment and risk profile associated with the pre-, during-, and post-pandemic phases. The detailed segmentation is as follows: (1) Full sample period (2012.3.2–2025.3.7): encompasses the pre-pandemic, pandemic, and post-pandemic phases; used for overall model training and comprehensive performance evaluation. (2) Pre-pandemic period (2012.3.2–2019.12.31): represents the normal functioning of China's capital markets under stable structural and policy conditions, serving as a crucial reference period for baseline model construction. (3) First year of the pandemic (2020.1.1–2020.12.31): characterized by the sudden outbreak of COVID-19, during which the capital market experienced extreme volatility. Public health-related firms drew intensive attention, leading to pronounced divergence in stock performance. This phase tests the model's adaptability under crisis conditions. (4) Second year of the pandemic (2021.1.1–2021.12.31): marked by the normalization of pandemic control and frequent policy adjustments, this period witnessed substantial transformation in business environments, providing a benchmark to assess the generalization ability of the predictive model. (5) Third year of the pandemic (2022.1.1–2022.12.31): featured continuous virus mutation and repeated shifts in control strategies, resulting in heightened industry uncertainty and complex market responses. This stage poses significant challenges to the stability of the prediction system. (6) Post-pandemic period (2023.1.1–2025.3.7): a stage of macroeconomic recovery and industrial revitalization, during which public health enterprises transitioned from emergency response to high-quality development. The capital market returned to fundamentals-driven logic, making this phase ideal for evaluating the model's effectiveness in “recovery markets” and its portfolio optimization capacity. Regarding the division of time periods, this study draws on the approaches of Lee et al. (54), Sobczak and Pawliczak (55), and Jin et al. (56), while making adjustments based on the specific trajectory of the COVID-19 outbreak and policy responses in China. When applying the Transformer model, 80% of the data is used as the training set, and the remaining 20% is designated as the testing set. The prediction results for different time periods are presented in Table 2.

**Table 2:** Evaluation of prediction results across different time periods.

**Period**	**Metric**	**MAE**	**MSE**	**RMSE**
Full sample	Mean	0.020745	0.000888	0.026975
	Std. dev.	0.010776	0.001110	0.012677
Pre-pandemic	Mean	0.024253	0.001270	0.031352
	Std. dev.	0.014244	0.002137	0.016954
First year of the pandemic (2020)	Mean	0.020021	0.000698	0.025269
	Std. dev.	0.006186	0.000416	0.007726
Second year of the pandemic (2021)	Mean	0.028439	0.001509	0.035700
	Std. dev.	0.015423	0.001547	0.015300
Third year of the pandemic (2022)	Mean	0.026021	0.001308	0.032811
	Std. dev.	0.015153	0.001547	0.015205
Post-pandemic	Mean	0.024433	0.001177	0.032693
	Std. dev.	0.008500	0.000792	0.010423

Table 2 presents the prediction results for stock returns of public health-related enterprises across different phases of the COVID-19 pandemic. The model's forecasting performance is evaluated using three standard error metrics—MAE, MSE, and RMSE—across distinct time periods, providing insight into how structural shifts in the capital market induced by public health events influence the model's accuracy and stability. During the full sample period (2012.3.2–2025.3.7), the Transformer model achieves relatively low prediction errors, demonstrating robust overall performance. The MAE is 0.020745, the MSE is 0.000888, and the RMSE is 0.026975, indicating a strong fitting capability. The model benefits from the long-term accumulation of structural features, which enhances its ability to identify temporal patterns. However, elevated volatility and market heterogeneity across the full period introduce a degree of uncertainty. The standard deviation values reveal substantial cross-sectional heterogeneity in prediction errors, suggesting that return volatility varies significantly across individual stocks. This highlights the presence of stock-specific dynamics and reinforces the importance of modeling approaches that can accommodate such variability.

During the pre-pandemic period (2012.03.02–2019.12.31), a phase characterized by economic stability, the model exhibits comparatively higher prediction errors, with an MAE of 0.024253, MSE of 0.001270, and RMSE of 0.031352. This counterintuitive result may be attributed to the relatively low market attention toward public health enterprises during that time, as the pricing mechanisms for this sector were influenced by multiple, often opaque, factors. In this normal period, public health concerns had not yet emerged as salient issues in the capital market, and the sector as a whole lacked distinct trend characteristics. This made risk forecasting more challenging and also exposed the insufficient capital support historically directed toward the public health system. The market operated with greater micro-level complexity, which made it difficult for the model to capture all relevant driving forces. The coexistence of long-term structural patterns and short-term noise likely limited the Transformer's capacity to effectively extract predictive signals from a market that was “stable but weakly directional.”

During the initial phase of the pandemic (2020.1.1–2020.12.31), the market underwent a rapid structural reconfiguration triggered by the sudden public health crisis, liquidity shocks, and widespread investor panic. Paradoxically, however, this period yielded the best model performance, with the lowest prediction errors: MAE of 0.020021, MSE of 0.000698, and RMSE of 0.025269. This counterintuitive improvement may be explained by the fact that, during the early stage of the pandemic, public health risk became the dominant market concern. The sudden outbreak led to intense market focus on healthcare and epidemic-prevention-related public health firms. Stock prices in this sector became closely aligned with the trajectory of virus transmission and policy signals, resulting in highly consistent price trends. The crisis period, in effect, increased information concentration and transparency—stock performance in the public health sector reflected both the firms' epidemic response value and their systemic importance. Rather than amplifying uncertainty, this phase reduced prediction ambiguity as the market entered a “structurally unified” state. The concentrated release of risk improved the model's ability to identify trend-following assets. The predictive stability observed during this phase provided both governments and investors with an effective window for early risk detection, thereby supporting precise resource allocation and timely policy interventions.

In the second year of the pandemic (2021.1.1–2021.12.31), prediction errors increased significantly. The MSE rose to 0.001509—the highest across all phases—while the MAE and RMSE reached 0.028439 and 0.035700, respectively, marking a clear deterioration in model performance compared to other years. The year 2021 represented a phase of normalized pandemic management. While vaccine deployment began and initial signs of macroeconomic recovery emerged, the market remained highly uncertain. Multiple global waves of infection and the emergence of virus variants intensified volatility in investor risk preferences. Shifts in policy direction affecting public health enterprises—such as turning points in vaccine company profitability and the withdrawal of subsidies for testing firms—altered valuation logics. At the same time, corporate earnings growth became increasingly uncertain. Overall, the frequent adjustment of health-related policies, ongoing transformation of firm fundamentals, and increased market complexity jointly contributed to a more difficult forecasting environment. Although the pandemic had not yet ended, the institutionalization of pandemic control paradoxically intensified market speculation on the long-term value of public health enterprises.

In the third year of the pandemic (2022.1.1–2022.12.31), the model's prediction errors showed a slight improvement compared to 2021, yet remained elevated. This persistent high level of error reflects continued market instability. Throughout 2022, the policy environment was marked by uncertainty—characterized by alternating periods of easing and tightening pandemic controls. In addition, external shocks such as geopolitical tensions and interest rate hikes by the U.S. Federal Reserve further exacerbated volatility in public health-related assets. Short-term market fluctuations remained frequent, indicating that even as the public health crisis approached its end, the financial system continued to experience “post-crisis” risk transmission effects.

In the post-pandemic period (2023.1.1–2025.3.7), macroeconomic conditions gradually recovered, and the public health sector transitioned from an emergency response phase to normalized governance. However, the market structure exhibited characteristics of a “new normal,” as capital began to reassess the long-term value of firms. Market pricing logic became increasingly rational, with restored earnings expectations emerging as the dominant factor in valuation. While the MAE remained comparable to the pre-pandemic period, the MSE showed a marked decline, indicating a reduction in extreme prediction errors and improved model stability. The RMSE suggests that overall prediction errors remained moderate, and forecasting accuracy nearly returned to pre-pandemic levels. These results imply that the model successfully adapted to the restructured market environment, demonstrating a degree of long-term generalization capability. Overall, the post-pandemic period was characterized by market restructuring, growing performance divergence among public health firms, and a return to rational capital allocation. Although prediction deviations did not improve dramatically, volatility clearly subsided. The model gradually regained its predictive ability in a more stable market, suggesting that the normalization of public health risk management contributed positively to financial system stability.

### 4.3 Investment performance comparison across different periods

To further explore the stage-specific impact of major public health events on capital market investment performance—particularly on public health-related enterprises—this study constructs long–short investment portfolios based on the 55 constituent stocks of the CSI Public Health Index. By integrating the pre-, during-, and post-pandemic subperiods, we systematically evaluate the evolution of investment returns and risk characteristics under different market environments. Building upon the return prediction results obtained using the Transformer model in the previous section, this analysis shifts focus to the portfolio level to examine the deeper implications of the pandemic shock on the public health sector. Specifically, we employ three performance metrics—annualized return, Sharpe ratio, and Sortino ratio—to assess portfolio outcomes from the perspectives of return magnitude, risk-adjusted performance, and downside risk control. Table 3 presents the portfolio return performance across the various pandemic phases.

**Table 3:** Portfolio returns across different periods.

**Period**	**Annualized average return**	**Sharpe ratio**	**Sortino ratio**
Full sample	0.226055	1.325372	2.037855
Pre-pandemic	−0.008501	−0.034368	−0.050146
First year of the pandemic (2020)	0.537138	2.856251	3.632034
Second year of the pandemic (2021)	−0.589107	−2.513340	−3.535249
Third year of the pandemic (2022)	−0.120103	−0.395913	−0.683095
Post-pandemic	−0.013514	−0.079009	−0.135693

From Table 3, the annualized average returns of the long–short portfolio exhibit notable variation across different periods. In 2020 (the initial phase of the pandemic), the annualized return reached 0.537138, the highest among all phases, reflecting substantial structural opportunities for excess returns in public health-related firms amid the outbreak. In contrast, returns sharply turned negative in 2021 and 2022 (−0.589107 and −0.120103, respectively), indicating that repeated policy shifts and sectoral restructuring led to heightened market turbulence. During the post-pandemic period (2023–2025), the portfolio showed marginal recovery, however, the return remained slightly negative at −0.013514. This suggests that, following a reversion to rational pricing mechanisms, short-term investment returns in the public health sector became relatively muted. The Sharpe ratio—a key indicator of risk-adjusted return—exhibits a trajectory broadly consistent with that of annualized returns. In 2020, the ratio peaked at 2.856251, reflecting the superior risk–return profile of public health-related assets amid heightened uncertainty. However, during the mid- to late-pandemic periods, the ratio turned negative, with a particularly sharp decline in 2021 to −2.513340, indicating a substantial increase in volatility and downside risk. Although there was a slight rebound in the post-pandemic phase (−0.079009), the overall performance remained weak, suggesting inadequate compensation for the risk undertaken. As shown in Table 3, the Sortino ratio peaked in 2020 at 3.632034, marking the highest value across all periods. This indicates that during the initial outbreak of the pandemic, certain public health-related companies—such as those involved in vaccine development, biomedical testing, and epidemic prevention equipment—experienced a surge in capital inflows, achieving high returns with relatively low downside risk. In contrast, the pre-pandemic (−0.050146) and post-pandemic (−0.135693) phases exhibited Sortino ratios close to zero, suggesting that investment portfolios during these periods lacked effective downside risk protection, and the return volatility was more difficult for investors to hedge. In 2021 (−3.535249) and 2022 (−0.683095), the Sortino ratios were negative, with 2021 marking the lowest point. This reflects a severe imbalance between returns and downside risks, driven by prolonged pandemic disruptions and recurrent policy shifts. The data highlight how excessive market responses to evolving risks severely undermined investment performance during these stages.

Overall, the trends observed across the three key investment performance metrics—annualized average return, Sharpe ratio, and Sortino ratio—demonstrate a high degree of consistency. The initial outbreak phase of COVID-19 in 2020 marked the strongest performance across all three indicators. During this period, the public health crisis triggered a rapid restructuring of the market, prompting concentrated capital flows into sub-sectors such as pharmaceutical manufacturing, epidemic prevention equipment, and biomedical testing. This surge was driven by a sharp increase in demand for pandemic-related assets, leading to pronounced upward price momentum and a heightened risk-averse sentiment among investors. These results suggest that sudden public health emergencies can create short-term opportunities for abnormal returns by amplifying structural pricing differentials, thereby offering a predictable pathway for crisis-period capital allocation and policy intervention.

In contrast, the mid-pandemic period (2021) represented the most vulnerable phase for the investment portfolio, with all three indicators experiencing steep declines. This deterioration reflects the compounding effects of policy uncertainty, viral mutations, and destabilized market expectations. During this stage, medical resource allocation reached a bottleneck, the initial vaccine-driven gains had been exhausted, and subsidies for diagnostic testing were being phased out. As a result, industry expectations became increasingly volatile, and investor confidence in public health-related enterprises deteriorated sharply. The substantially negative returns during this period illustrate how mid-stage public health crises can transmit uncertainty risks more deeply into financial markets, exacerbating systemic fragility.

Although the late-pandemic phase (2022) showed slight improvements across all indicators compared to the previous year, the values remained low or negative. These figures indicate that markets had not yet established a stable anchor between pandemic control measures and shifting policy regimes. The public health sector—particularly pharmaceuticals and healthcare services—was still undergoing a valuation correction, and the strategic direction of the industry had yet to be redefined. This period underscores the need for consistent and forward-looking industrial guidance from regulators, rather than reactive policy swings that may further destabilize investor sentiment.

Post-pandemic, the market entered a phase of gradual recovery. Although the annualized return during this period remained slightly negative at −0.013514, volatility declined significantly, suggesting that investor expectations were becoming more rational and that the market was slowly reverting to pre-pandemic conditions. Over the entire sample period, the annualized return reached 0.226055, highlighting the long-term viability of structurally informed investment strategies in navigating public health crises. This result provides empirical evidence that constructing investment frameworks around the evolution of public health emergencies can enhance risk management capabilities and mitigate the asset losses associated with systemic shocks.

### 4.4 Cumulative return analysis

**Figure 1:**
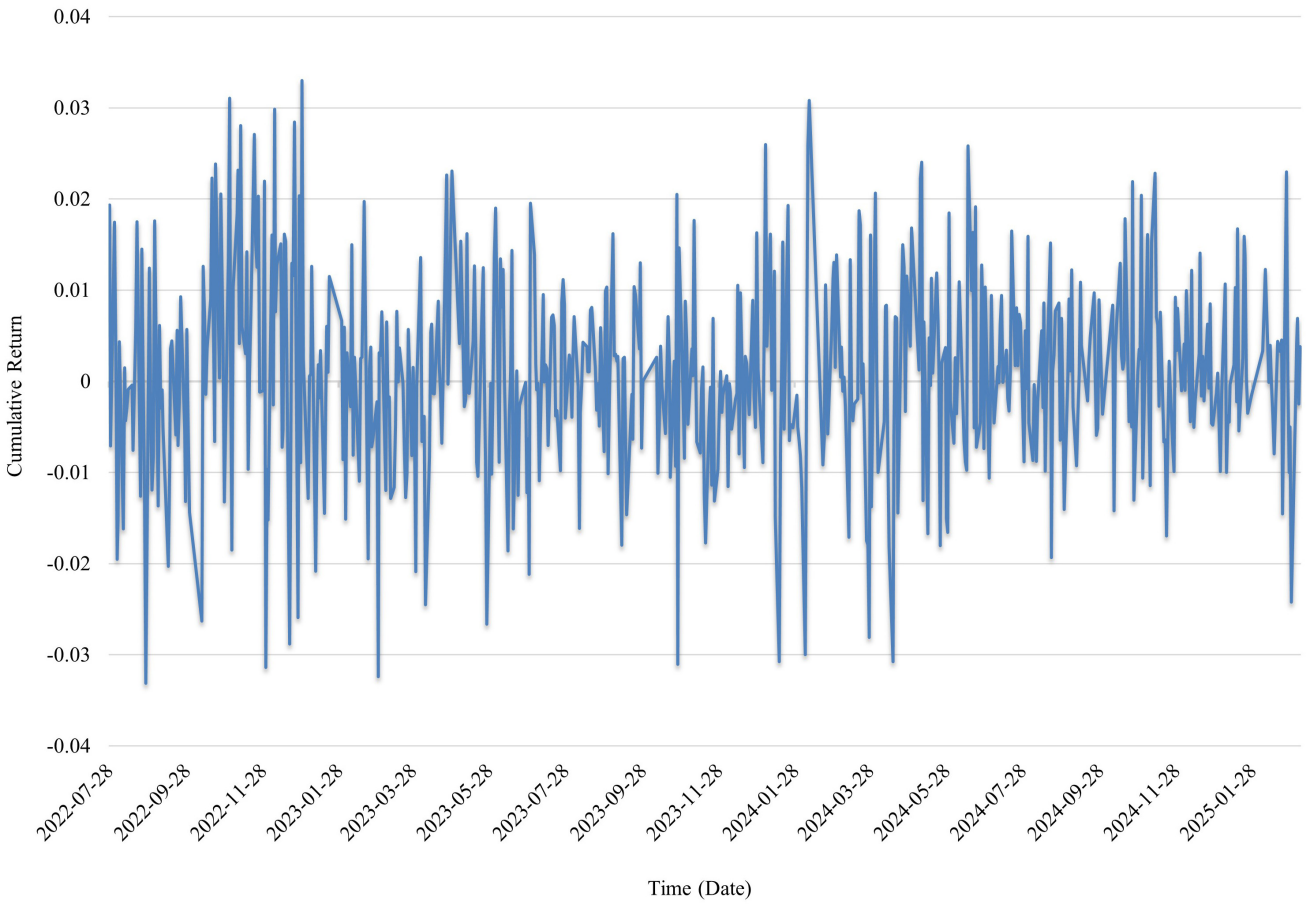
Cumulative returns over the full sample period.

To provide a more intuitive understanding of the performance of the long-short investment portfolio constructed based on the Transformer model's predicted returns, this study presents cumulative return line charts across different periods. These visualizations are interpreted in the context of the evolution of the public health crisis and corresponding capital market dynamics. Figure 1 illustrates the cumulative return trajectory of the investment portfolio formed using the full-sample prediction results. The horizontal axis represents the trading dates, while the vertical axis denotes the cumulative return level of the portfolio. This visualization serves to highlight the temporal fluctuations in investment performance and enables a phase-specific comparison of profitability and resilience under varying market conditions induced by the COVID-19 pandemic.

Figure 1 illustrates the cumulative return performance of the long-short investment portfolio constructed using Transformer model predictions, trained on the full sample dataset and applied to public health sector stocks during the 2022–2025 period. In 2022, the portfolio exhibited significant return fluctuations, reflecting heightened market sensitivity and volatile investor expectations amid frequent adjustments to pandemic-related policies. As public health measures were gradually optimized and the post-pandemic economy began to recover, the market performance of public health enterprises became more stable, indicating a progressive return of investor focus to fundamental values. Overall, the portfolio effectively captured the risk release and structural revaluation processes of public health-related firms during the late stages of the pandemic. The sector experienced pronounced structural volatility in the aftermath of the public health crisis, however, as the outbreak subsided, market sentiment gradually stabilized and investment valuations became more rational. The long-short strategy proved capable of identifying short-term mispricings and long-term trends, offering robust support for risk monitoring and capital allocation in the public health sector. These findings underscore the strategy's practical relevance for enhancing risk control and investment decision-making during and after systemic health events.

**Figure 2:**
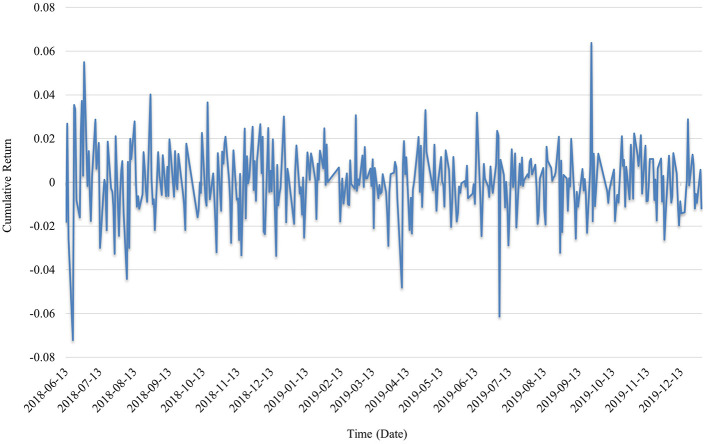
Cumulative returns during the pre-pandemic period.

In Figure 2, the cumulative return curve during the pre-pandemic period exhibits high-frequency fluctuations without a clear upward or downward trend. Overall portfolio returns oscillate around the zero axis, indicating a relatively stable performance. The few instances of sharp fluctuations are primarily associated with liquidity events or positive sector-specific expectations. The maximum single-day gain is ~0.06, while the maximum loss exceeds −0.07, suggesting that during normal market conditions, the public health sector did not receive systematic attention or speculative inflows, and its risk–return profile remained neutral. Market sentiment was generally subdued, and valuation differentiation within the sector was not yet pronounced, reflecting the limited systemic importance attributed to public health enterprises by capital markets at that time. The shape of the curve is consistent with the previously observed negative values of annualized return, Sharpe ratio, and Sortino ratio for the same period.

**Figure 3:**
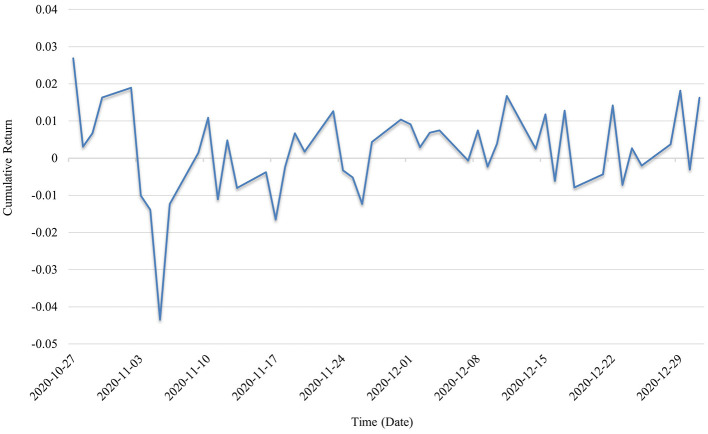
Cumulative returns in 2020.

In the first year of the outbreak (2020), as shown in Figure 3, the cumulative return curve derived from the Transformer model's predictions displayed a generally upward trend accompanied by substantial volatility. The portfolio experienced a sharp single-day drop exceeding −0.04, reflecting heightened market panic. Throughout this initial pandemic year, rising informational uncertainty, frequent phase-specific adjustments to containment policies, and the rapid shifts in market sentiment led to pronounced short-term fluctuations in the valuations of public health–related firms. Nevertheless, the relatively stable yet oscillating upward movement indicated sustained investor confidence in the long-term allocation to public health assets. The year 2020 marked the first full-scale outbreak of COVID-19, during which public health–related enterprises became the focal point of capital market attention. Significant capital inflows were directed toward sectors such as vaccine development, diagnostic services, and protective equipment, driving rapid valuation increases for certain companies and generating stronger trend-following trading signals.

**Figure 4:**
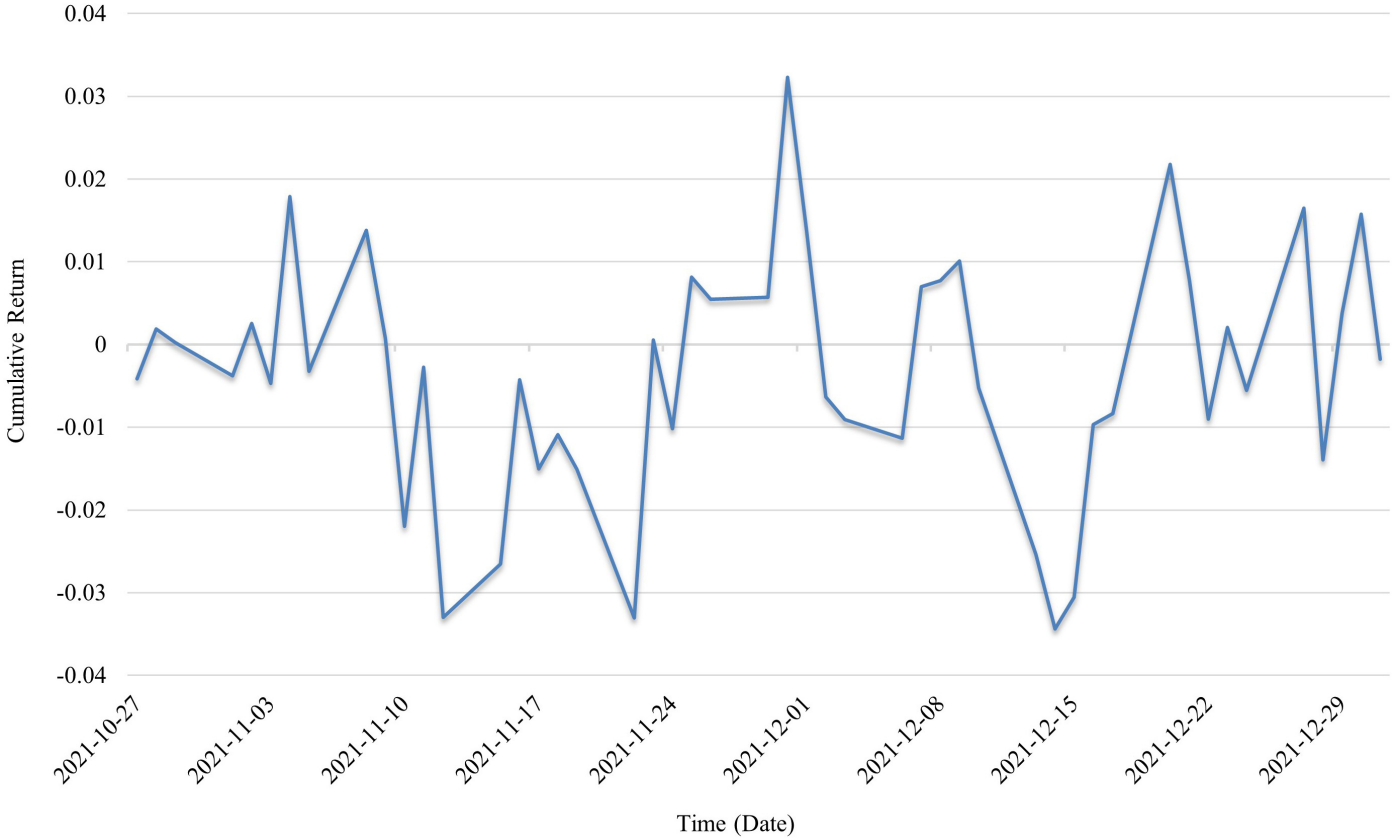
Cumulative returns in 2021.

In the second year of the pandemic (2021; as shown in Figure 4), cumulative returns exhibited intense high-frequency fluctuations, with significantly expanded oscillation ranges. Compared with the trend recovery pattern observed in 2020, the 2021 curve took on a “sawtooth” shape, indicating that the sector was caught between policy disturbances and shifting market expectations. During this period, some firms began to face pressure to deliver actual performance, while factors such as medical supply surpluses and adjustments to pandemic prevention policies led to a sharp divergence in investor sentiment. Although structural opportunities still existed, the increased difficulty of identification highlighted the importance of quantitative investment strategies in risk recognition.

**Figure 5:**
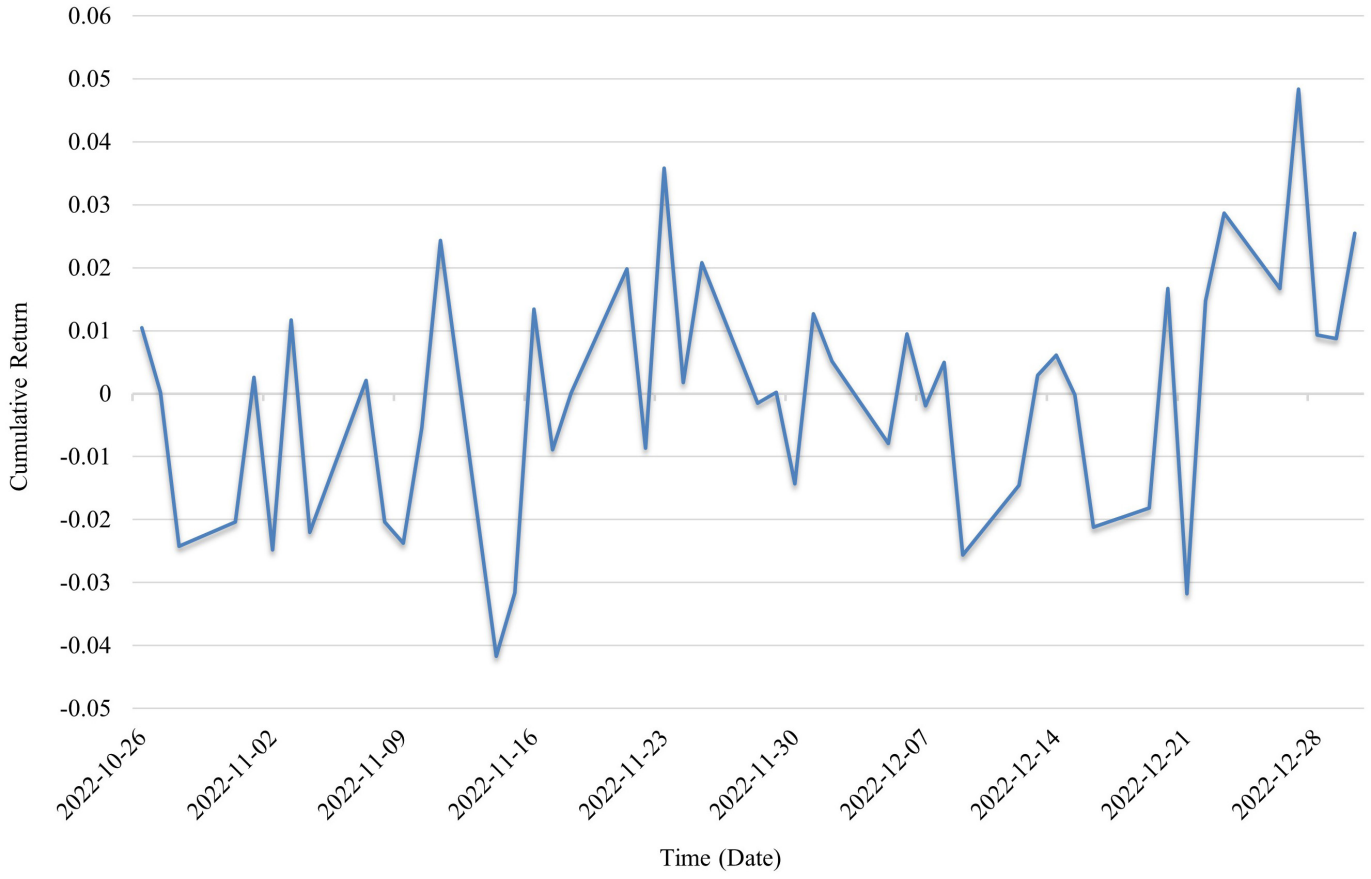
Cumulative returns in 2022.

In the third year of the pandemic (2022; as shown in Figure 5), the cumulative return range of the public health sector investment portfolio expanded significantly. The overall return curve lacked a clear direction, featuring alternating spikes and sharp pullbacks, reflecting unstable market sentiment and the dominance of news-driven movements. Some pandemic-related firms benefited temporarily from policy subsidies or expectations of supply shortages, resulting in short-term surges. In contrast, traditional healthcare service companies faced performance pressure and demand uncertainty. Policy shifts became a key variable, with stock prices showing particularly sharp short-term volatility during transitions, such as from the “zero-COVID” strategy to “targeted prevention and control.”

**Figure 6:**
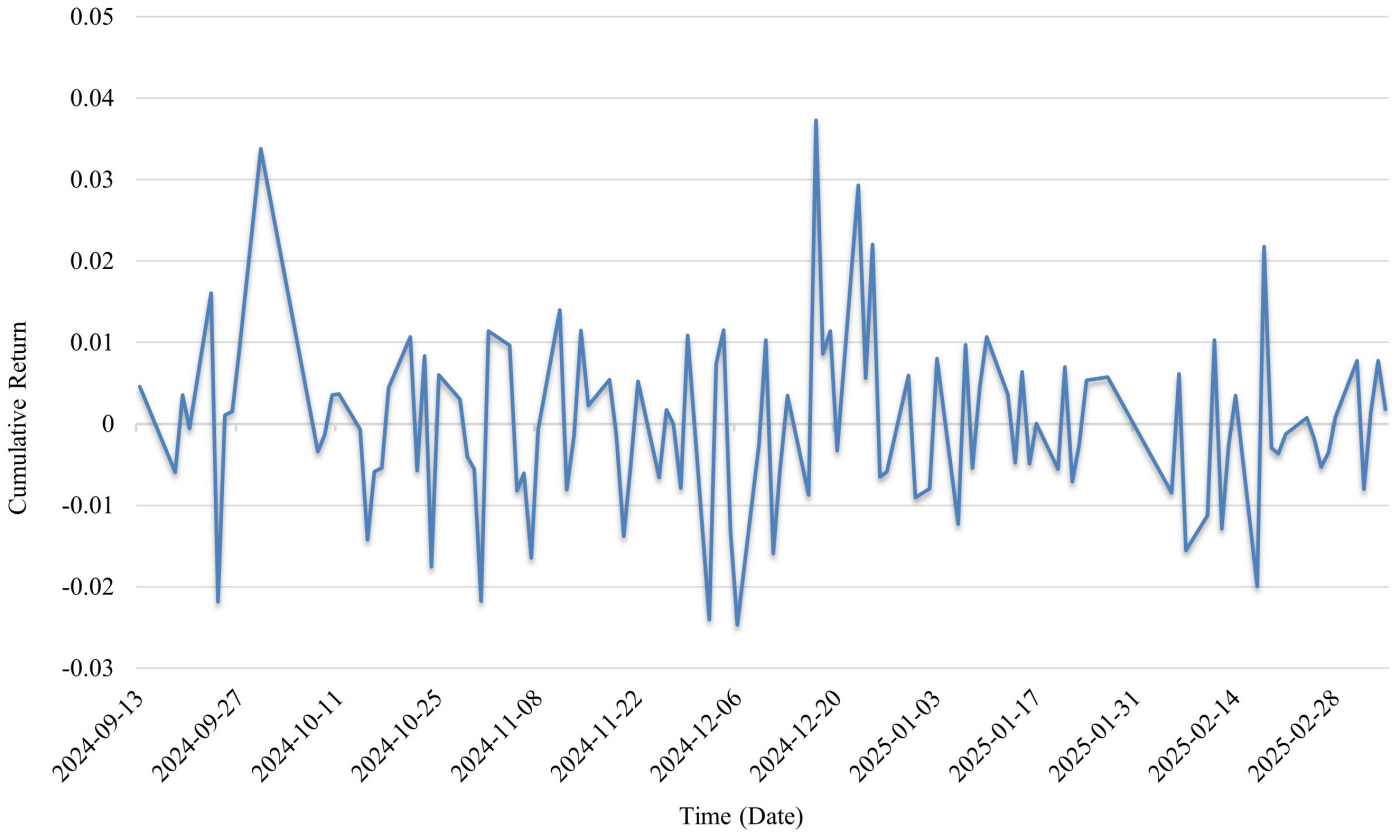
Cumulative returns in the post-pandemic period.

In Figure 6, during the post-pandemic period (2023–2025), the cumulative return curve remains generally flat with narrowed fluctuations, with most returns concentrated around the ±0.01 range, indicating reduced market volatility. The market valuation of public health-related firms tends to become more rational in the post-pandemic phase, investor expectations gradually stabilize, and short-term speculative sentiment significantly weakens. The industry has transitioned from an early stage dominated by sentiment to a steady state primarily driven by fundamentals.

### 4.5 Effectiveness analysis

**Figure 7:**
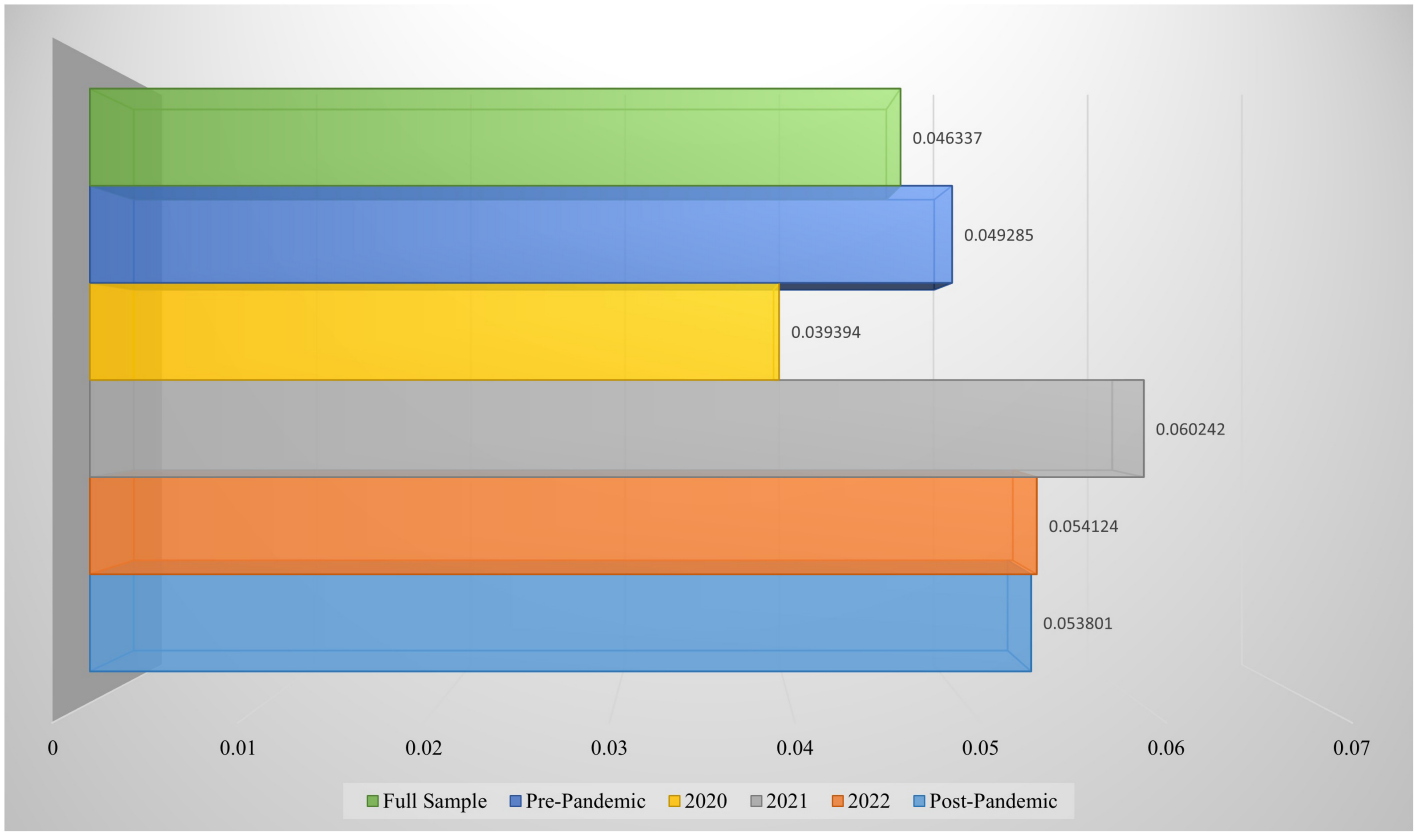
Evaluation of SVM prediction results.

To validate the reliability and robustness of the findings, this study further conducts a comparative analysis using the support vector machine (SVM) model, employing the same dataset and time period segmentation as in the primary analysis. As a classical machine learning technique, SVM has been extensively applied in financial market research—for instance, in stock market prediction studies by Bazrkar and Hosseini (57), Kuo and Chiu (58), and Long et al. (59). In this analysis, the prediction performance of the SVM model is evaluated using the root mean square error (RMSE), while investment performance is assessed using the Sharpe ratio. Consistent with the main analysis, the evaluation is conducted not only on the full sample period but also across five distinct sub-periods, corresponding to different stages of the COVID-19 pandemic and their associated impacts on the market conditions faced by public health–related enterprises. The prediction results of the SVM model are presented in Figure 7.

As illustrated in Figure 7, the SVM model yielded an RMSE of 0.046337 over the full sample period, indicating a reasonable level of adaptability in long-term forecasting. The lowest RMSE, 0.039394, occurred during the initial outbreak of the COVID-19 pandemic in 2020, suggesting that the model effectively captured the strong deterministic patterns driven by industry-specific characteristics and the direct impact of the public health crisis amid a period of rapid structural changes in the market. In 2021, the RMSE increased to 0.060242—the highest among all sub-periods. This stage was characterized by normalized pandemic management, fluctuating public health policies, and volatile market expectations, which collectively amplified valuation instability and hindered the SVM's ability to capture complex signals under heightened volatility. The RMSE slightly declined to 0.054124 in 2022 but remained at an elevated level, reflecting continued heterogeneity among firms in the sector due to ongoing policy and environmental uncertainties, with market conditions yet to return to full stability. The RMSE for the pre-pandemic period was marginally higher than that of the full sample but lower than that during the mid-pandemic stages, suggesting that under relatively stable macroeconomic conditions, the SVM retained a basic level of predictive competence. In the post-pandemic period, although market volatility had subsided (RMSE = 0.053801), structural changes continued to affect prediction accuracy. During this phase, the overall market stabilized, yet investor behavior and corporate operational logic underwent significant transformations, weakening the forecasting power of traditional methods such as SVM.

**Figure 8:**
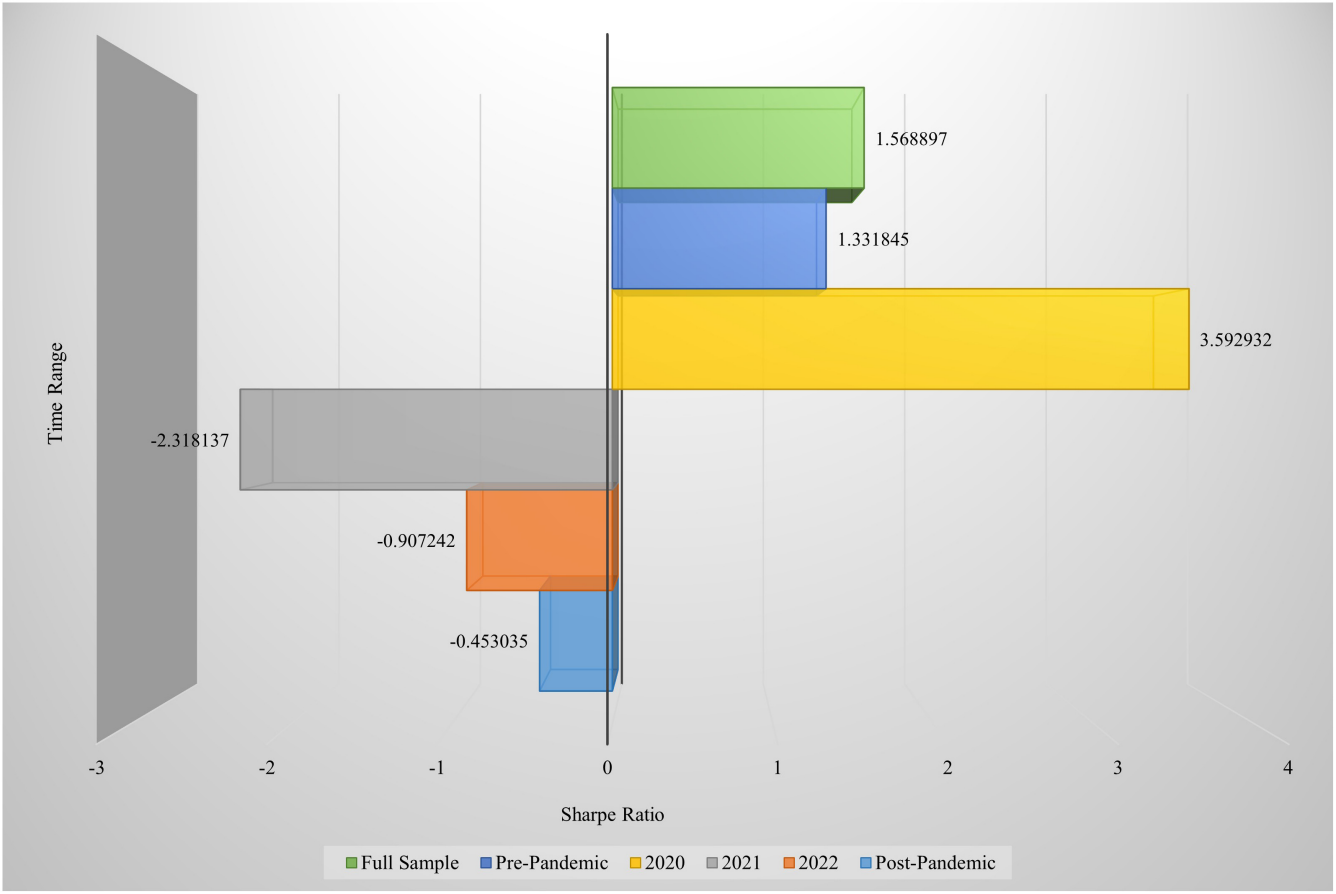
Sharpe ratios of investment portfolios based on the SVM model.

In summary, the SVM model demonstrated superior performance during the initial phase of the pandemic, confirming the concentration and identifiability of market signals in the early stages of a public health crisis. However, the increase in prediction error during the mid- and post-pandemic periods indicates that, as industry structures became more complex and policy uncertainties intensified, traditional models like SVM struggled to perform effective risk assessments under dynamic conditions. From the perspective of capital market risk management, these findings suggest that in response to sudden public health emergencies, models with strong capabilities in high-dimensional feature extraction and nonlinear fitting—such as the Transformer—should be prioritized to enhance risk identification and response capacity. Compared to the SVM, the Transformer consistently outperformed across all time periods in terms of predictive accuracy. Furthermore, to assess the impact of major public health events on the market performance of health-related enterprises, we constructed long-only investment portfolios for different time periods. The Sharpe ratios of long-only portfolios based on the SVM model are presented in Figure 8.

As shown in Figure 8, the Sharpe ratio for the full sample period is 1.568897, indicating that the long-only portfolio constructed using the SVM model achieved strong risk-adjusted returns over the long term, maintaining stable performance throughout multiple waves of pandemic shocks and recovery cycles. The pre-pandemic Sharpe ratio stood at 1.331845, suggesting that, prior to the outbreak, public health–related enterprises operated in a context of stable market expectations and reasonable risk premiums. During this period, the model effectively captured fundamental industry signals and long-term valuation trends, yielding solid investment returns. In 2020, at the onset of the pandemic, the Sharpe ratio peaked at 3.592932—the highest among all periods—reflecting a phase of structural repricing in the capital market triggered by the public health emergency. Leading enterprises in pandemic-related sectors realized substantial excess returns, and the SVM model successfully identified these trend-driven opportunities and delivered high risk compensation. However, the strategy broke down in 2021, with the Sharpe ratio plunging to −2.318137, indicating severe underperformance. This period was marked by heightened uncertainty under normalized pandemic control, frequent policy shifts, and repeated valuation adjustments, all of which rendered the model's signals ineffective and led to large losses, with risks significantly outweighing returns. In 2022, the Sharpe ratio slightly recovered to −0.907242, yet remained in negative territory, signaling that the market had not yet fully adapted to the evolving policy rhythm or valuation logic within the public health sector. Investor confidence remained weak, and health-related stocks lacked clear upward trends. In the post-pandemic period, the Sharpe ratio further improved to −0.453035 but failed to return to positive levels. During this stage, earnings expectations for public health firms normalized, and the absence of new policy catalysts or heightened investor attention led to subdued profitability in a low-volatility environment.

This analysis further reveals how public health crises reshape the risk–return dynamics in capital markets. In the early phase of the pandemic, the sector benefited from intense policy support and market attention, giving rise to a pronounced structural uptrend, with the model's predictive accuracy and investment performance reaching their peak. However, in the mid-to-late stages of the pandemic, as policies tightened or were phased out, market uncertainty intensified, and corporate earnings became more volatile, the model's investment strategy deteriorated significantly, exposing substantial downside risk. The robustness analysis confirms that, consistent with the results from Transformer-based portfolio construction, major public health events exert phase-dependent impacts on the investment performance of public health–related firms in capital markets.

## 5 Conclusion

This study, set against the backdrop of the COVID-19 pandemic as a major public health shock, selects 55 listed companies from the CSI Public Health Index as the research sample. Utilizing the Transformer deep learning model to predict stock returns, the study further constructs long-short investment portfolios to systematically evaluate investment value variations across pre-pandemic, pandemic, and post-pandemic periods. By comparing prediction error metrics, investment performance indicators, and cumulative return trajectories across distinct phases, this research reveals the structural evolution of public health-related enterprises under public health shocks and assesses the effectiveness and limitations of deep learning strategies in adapting to varying market environments. Special attention is paid to the dynamic evolution of capital market structures and firm performance triggered by major health events, aiming to deepen the understanding of risk response mechanisms in public health-related industries from an economic and financial perspective.

Specifically, public health events have significantly altered market structures, creating phase-specific trend investment opportunities. During the initial outbreak in 2020, public health-related firms became the focal point of market attention. The Transformer model effectively captured trend signals and enabled efficient trading decisions, yielding low prediction errors—MAE of 0.020021, MSE of 0.000698, and RMSE of 0.025269. The corresponding investment portfolio achieved outstanding performance, with an annualized return of 0.537138, a Sharpe ratio of 2.856251, and a Sortino ratio of 3.632034. The cumulative return curve exhibited a clear upward trend, demonstrating that public health-related stocks under structural shocks possess strong predictability and significant excess return potential. In the mid-to-late stages of the pandemic, heightened market uncertainty led to diminished model stability and profitability. From 2021 to 2022, portfolio returns deteriorated markedly, with increased volatility and frequent drawdowns in cumulative returns. This reflects substantial fluctuations in earnings expectations and a loosening of capital structures driven by intensive policy adjustments and heightened risk exposure. These findings indicate that under repeated policy shifts, volatile market structures, and uncertain profit forecasts for public health firms, the model's ability to extract reliable signals and maintain robustness was severely challenged. In the post-pandemic period, the market gradually returned to rationality. Although the strategy's volatility subsided and predictive stability improved between 2023 and 2025, both annualized returns and risk-adjusted performance indicators remained low. This suggests that the industry is undergoing a revaluation process, transitioning from a “pandemic-driven logic” to one grounded in long-term fundamentals. While the model has begun to adapt to the new market structure, it has yet to establish a stable profit-generating mechanism under this “new normal.” The effectiveness analysis based on the SVM model provides additional empirical support for the robustness of the study's conclusions.

The market performance of public health enterprises is closely linked to macroeconomic policies and industry structure, with model-based strategies demonstrating a comparative advantage during phases characterized by strong trends. Empirical evidence shows that at different stages, public health-related firms are jointly influenced by pandemic developments, fiscal support, and regulatory adjustments, exhibiting significant structural heterogeneity. Under conditions of clear policy direction and well-defined sectoral differentiation, deep learning-based strategies display robust pattern recognition capabilities. Conversely, during periods of intense policy uncertainty or structural market transitions, the robustness and stability of model predictions are notably constrained. Given the increasing frequency of public health emergencies and the elevation of health security to a national strategic priority, it is imperative to explore the behavioral logic of public health enterprises within financial markets. Revealing the structural evolution of the sector in response to exogenous shocks can help identify the systemic impact of public health events on capital markets. Unlike traditional studies that assess the pandemic's effects from a macroeconomic perspective, this paper draws on firm-level data and capital market performance to present a quantitative framework for identifying structural risks and forecasting asset trends. This approach offers both theoretical and practical value for understanding how capital markets operate during crisis periods and how investment strategies adapt in the face of unforeseen disruptions. For investors, the stage-dependent predictability observed in public health–related enterprises during different phases of the pandemic suggests that portfolio strategies should be dynamically adjusted in response to the evolution of the pandemic and related policy shifts. For policymakers, AI-based forecasting models such as the Transformer can facilitate the early identification of sectoral risks, support timely intervention, improve the efficiency of resource allocation, and enhance the resilience of public health systems and the stability of financial markets.

In conclusion, the Transformer model demonstrates considerable potential in forecasting returns and constructing investment strategies for public health–related enterprises, particularly in trend-driven markets triggered by sudden events. Its strong capability in capturing directional movements makes it a promising tool under such conditions. However, the model encounters limitations during periods characterized by structural complexity and heightened policy uncertainty. This study contributes data-driven empirical evidence for understanding the transmission mechanisms of public health crises in capital markets and offers methodological insights for developing more robust and responsive investment and risk identification frameworks. Nonetheless, several limitations remain. Future research could incorporate a broader set of external features and macroeconomic factors to enhance the model's representational capacity under complex public health scenarios. Additionally, integrating the Transformer model with approaches such as graph neural networks or reinforcement learning may further improve its adaptability and stability in highly uncertain market environments. Expanding the research scope to include other crisis-sensitive industries or international markets would also enable cross-country comparisons and a deeper understanding of how public health risks affect capital markets under different institutional settings, thereby enhancing the external validity and policy relevance of the findings.

## Data Availability

The original contributions presented in the study are included in the article/supplementary material, further inquiries can be directed to the corresponding author.
